# CD11c+ antigen presenting cells from the alveolar space, lung parenchyma and spleen differ in their phenotype and capabilities to activate naïve and antigen-primed T cells

**DOI:** 10.1186/1471-2172-9-48

**Published:** 2008-08-13

**Authors:** Kapilan Kugathasan, Elizabeth K Roediger, Cherrie-Lee Small, Sarah McCormick, Pingchang Yang, Zhou Xing

**Affiliations:** 1 Department of Pathology and Molecular Medicine, Centre for Gene Therapeutics, McMaster University, Hamilton, Ontario, Canada; 2 M.G. DeGroote Institute for Infectious Disease Research, McMaster University, Hamilton, Ontario, Canada

## Abstract

**Background:**

The lung is divided into two major compartments: the alveolar space and the parenchyma. The alveolar macrophages are the first line of leukocytes in the lung taking up incoming microbes or microbial antigens whereas the parenchymal dendritic cells (DCs) are believed to be the sole potent antigen presenting cells (APCs) in the lung. Both resting alveolar macrophages and parenchymal DCs express CD11c. Several important questions remain to be elucidated: 1] to which extent the alveolar space and lung parenchymal CD11c+ APCs differ in their phenotype and ability to activate naïve T cells; 2] whether they differ in their ability to activate antigen-experienced or -primed T cells; and 3] whether these lung CD11c+ APC populations differ from the splenic CD11c+ APCs which have been commonly used for understanding APC biology.

**Results:**

CD11c+ APCs from the alveolar space, lung parenchyma, and the spleen display differential co-stimulatory molecule expression and cytokine responsiveness upon stimulation. Alveolar space APCs are weak activators of naïve T cells compared to lung parenchymal and splenic CD11c+ APC populations. However, alveolar space APCs are able to potently activate the in vivo microbial antigen-primed T cells to a similar extent as lung parenchymal and splenic APCs.

**Conclusion:**

Together our findings indicate that alveolar CD11c+ APCs have a specialized T cell-activating function, capable of activating antigen-primed, but not naïve, T cells whereas lung CD11c+ APCs are capable of activating both the naïve and antigen-primed T cell populations.

## Background

The lung is a mucosal organ that is continuously exposed to the outside environment, rendering it one of the major sites of primary bacterial and viral infections. It is generally believed that upon pathogenic microbial invasion, antigen presenting cells (APCs) such as dendritic cells (DCs) and macrophages present in the lung intercept these microbes and participate in the initiation of ensuing innate and adaptive T cell responses. However, the lung is divided into two major compartments: the alveolar space and the parenchyma. In the steady state, the vast majority of cells in the alveolar space, which can be harvested by bronchoalveolar lavage (BAL), are alveolar macrophages (AMs), which are the first line of leukocytes in the lung taking up incoming microbes or microbial antigens [1-4]. On the other hand, the lung parenchymal tissue including the airway epithelium comprises a variety of APC types including DCs and B cells [5,6]. Traditionally, it is the lung DCs that are deemed the most critical to transporting antigens from tissue to the local draining lymph nodes to activate naïve T cells [7,8].

The issue regarding whether alveolar macrophages are capable of naïve T cell activation has remained controversial [2,4,9-15]. While AMs are generally thought to be poor T cell stimulators or even immunosuppressive as shown in some studies [11,16,17], they have also been shown to be able to activate certain T cell subsets [9]. The possibility for AMs to act as functional APCs is further supported by the evidence that macrophages could migrate to the local lymphoid tissues or acquire DC characteristics [13,18-20]. Furthermore, local non-migratory residential APCs including macrophages are thought to play a role in presenting antigen to and activating antigen-experienced or effector/memory T cells within the lung parenchyma [21]. In comparison, there has been well-established evidence to support an indisputable role by lung parenchymal DCs in transporting antigens to the secondary lymphoid tissue and activating naïve T cells [22-24]. However, several important questions remain to be elucidated: 1] to which extent the alveolar space CD11c+ APCs and lung parenchymal CD11c+ APCs differ in their phenotype and ability to activate naïve T cells; 2] whether they differ in their ability to activate antigen-experienced or -primed T cells; and 3] whether these lung CD11c+ APC populations differ from the splenic CD11c+ counterparts which have been often used for understanding APC biology. The lack of such knowledge is due to the fact that the majority of studies have thus far examined the phenotypes and functions of APC populations from various tissue compartments in isolation [25-28] or the direct comparative studies were carried out by using bone marrow- or spleen-derived macrophages and DCs [29-31]. Emerging evidence suggests that the knowledge generated at one site may not apply to another site as different anatomical locations or tissue microenvironments profoundly influence the phenotype and function of APC populations [5,32-34]. The knowledge regarding the relative T cell activation capacities of APCs within different tissue compartments will help us understand the immune regulatory mechanisms and develop APC-based strategies for immune manipulation.

In the present study, by using various approaches we have directly compared the phenotype of alveolar space CD11c+ APCs and the lung parenchymal CD11c+ APCs and their capability to activate both naïve and antigen-primed T cells. We have also compared these lung CD11c+ APC populations with the splenic CD11c+ APCs. We report that CD11c+ APCs from the alveolar space, lung parenchyma, and the spleen display differential co-stimulatory molecule expression and cytokine responsiveness. In addition, alveolar space APCs are poor activators of naïve T cells compared to lung parenchymal and splenic CD11c+ APC populations. However, alveolar APCs are able to potently activate the in vivo microbial antigen-primed T cells to a similar extent as lung parenchymal and splenic CD11c+ APCs.

## Results

### Freshly isolated CD11c+ APC populations from the alveolar space, lung parenchyma, and spleen express differential levels of co-stimulatory molecules

**Figure 1:**
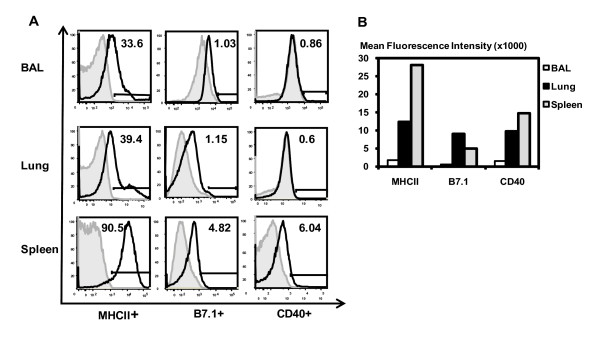
**Co-stimulatory molecule expression of CD11c+ APCs from the alveolar space, lung parenchyma, and the spleen.** Surface co-stimulatory molecule expression was analyzed by gating on CD11c+ cell populations by FACS. A, representative histograms showing the percentage of co-stimulatory molecules expression by CD11c+ cells. Solid lines, specific staining; shaded histograms, appropriate isotype controls. B, Average Mean Fluorescent Intensity (MFI) of co-stimulatory markers. Data are representative of three independent experiments.

Alveolar macrophages (AMs) in the lung are well known to express the cell surface marker CD11c which is commonly expressed by murine DC populations in other tissue sites such as the lung interstitium and spleen [22,35-38], thus resulting in the vast majority (> 95%) of AMs in naïve lungs being CD11c+ [39,40]. This fact has allowed us to conveniently isolate AM and lung interstitial and splenic DC populations based on CD11c selection and subsequently compare their phenotypes and functions. Thus, by using this approach, we first compared the co-stimulatory molecule expression of CD11c+ APC populations freshly isolated by MACS purification from the alveolar space, lung parenchyma and the spleen as expression of co-stimulatory molecules on APCs is critical to T cell activation. Since the alveolar space APCs were isolated first by bronchoalveolar lavage (BAL) before subjecting to MACS purification, these APCs were also called BAL APCs. We found that similar percentages of BAL and lung parenchymal CD11c+ APCs expressed major histocompatibility class II (MHC II) molecules, B7.1 and CD40 molecules on their surface (Figure 1A). In comparison, a greater proportion of splenic CD11c+ APCs expressed these molecules (Figure 1A). However, the mean fluorescence intensity (MFI) reveals that on average the amount of co-stimulatory molecules present on the BAL CD11c+ cells is much less than on the lung parenchymal CD11c+ cells while the splenic CD11c+ cells expressed the greatest MFI of these molecules (Figure 1B). Thus, CD11c+ cell populations located in different anatomical compartments express differential levels of co-stimulatory molecules on their surface, suggesting that they may also have differential functions or effector activities.

### Differential levels of cytokine production by CD11c+ APC populations from the alveolar space, lung parenchyma and spleen upon stimulation

**Figure 2:**
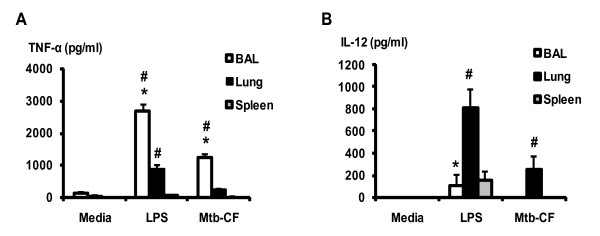
**Proinflammatory cytokine production by freshly isolated CD11+ APCs upon stimulation.** Freshly isolated CD11c+ APCs from the BAL, lung parenchyma, and the spleen were cultured for 48 h with LPS or mycobacterial antigens (Mtb-CF). Culture supernatants were measured for TNF-α (A) and IL-12 (B) by ELISA. Results are representative of three independent experiments and are presented as mean ± SD. *p < 0.05 compared to the corresponding lung data. #p < 0.05 compared to the corresponding spleen data.

Having observed differential co-stimulatory molecule expression by CD11c+APCs from the BAL, lung parenchyma and the spleen, we next evaluated the cytokine responses of these APC populations. To this end, cells were cultured for 48 h with either LPS or mycobacterial antigens. Using ELISA, we elected to look at the pro-inflammatory cytokine TNF-α and the type 1 cytokine IL-12 as the both are considered the major players in the activation and regulation of type 1 T cell responses and the maintenance of granulomatous responses against intracellular pathogens [41]. While BAL CD11c+ APCs produced significantly greater amounts of TNF-α in response to both LPS and Mtb-CF stimulation than both lung parenchymal CD11c+ and splenic CD11c+ APCs (Figure 2A), splenic CD11c+ APCs produced the least amounts of this cytokine. On the other hand, lung parenchymal APCs produced the greatest amounts of IL-12 in response to both LPS and Mtb-CF, whereas airway and spleen APCs produced barely detectable amounts of IL-12 (Figure 2B). These APC populations all produced undetectable levels of anti-inflammatory cytokine IL-10 (data not shown). Thus, in addition to phenotypic differences, airway luminal, lung parenchymal, and splenic CD11c+ APCs produce differential levels of type 1 cytokines in response to LPS or mycobacterial antigens stimulation.

### Alveolar space CD11c+ APCs are poor stimulators of allogeneic T cells compared to splenic CD11c+ APCs

**Figure 3:**
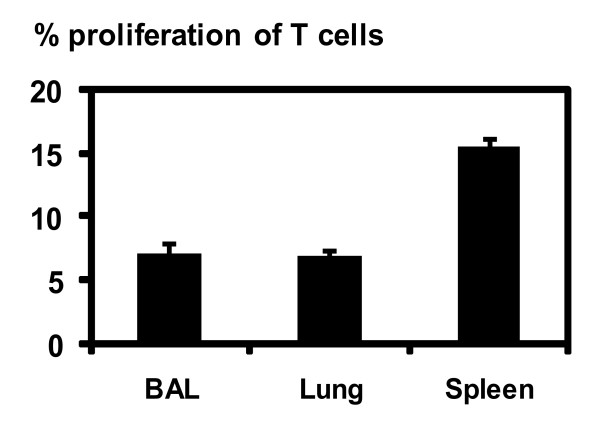
**Allogeneic T cell proliferation stimulated by CD11c+ APCs isolated from BAL, lung parenchyma and spleen.** Freshly isolated BAL, lung, and splenic CD11c+ APC populations from C57Bl/6 mice were co-cultured with CFSE-labelled splenic T cells from Balb/c mice. The extent of allogeneic T cell proliferation was analyzed by FACS. Values shown are subtracted from appropriate controls and are the means ± SD of triplicate samples, representative of two independent experiments.

Since we observed differential co-stimulatory molecule expression and cytokine responses by CD11c+ cells from the three tissue compartments, we set out to examine and compare their capacity in T cell activation. We first examined their ability to stimulate allogeneic T cell proliferation. To this end, a mixed leukocyte reaction (MLR) was carried out by using CD11c+ cells from the BAL, lung parenchyma, and spleen of C57Bl/6 mice in a co-culture system at different ratios with CFSE labelled T cells isolated from Balb/c spleens. A ratio of 1:5 APC vs. T cells yielded the optimal amount of proliferation in our system. In keeping with their similar levels of MHC class I and II molecule expression (Figure 1), both BAL and lung interstitial APCs stimulated similar MLR (Figure 3). In comparison, the splenic APCs triggered greater levels of MLR (Figure 3), consistent with their higher levels of expression of MHC and co-stimulation molecules (Figure 1).

### Differential levels of naïve antigen-specific transgenic T cell activation by peptide-loaded CD11c+ APC populations from the alveolar space, lung parenchyma and spleen

**Figure 4:**
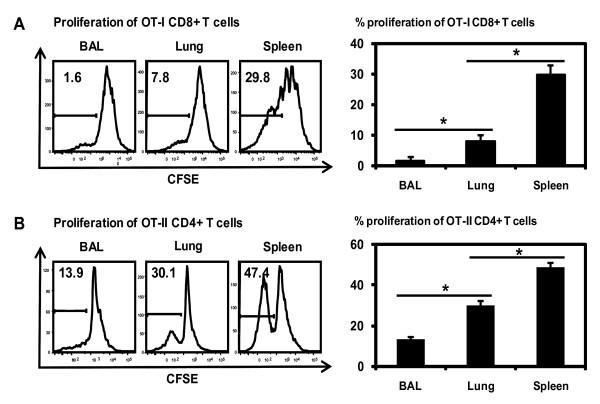
**Antigen-specific transgenic T cell activation by peptide-pulsed CD11c+ APCs from BAL, lung parenchyma and spleen.** CD11c+ APC populations isolated from BAL, lung and spleen were pulsed with OVA OT-I (SIINFEKL) or OT-II peptide and co-cultured for 48 hours with CFSE-labelled OT-I CD8+ T cells or for 72 hours with CFSE-labelled OT-II CD4+ T cells. Transgenic T cell proliferation was analyzed by FACS. A, OT-I CD8 T cell proliferation. B, OT-II CD4 T cell proliferation. Representative histograms of T cell proliferation are shown. The average T cell proliferation rates are shown in bar graphs after subtracting from appropriate controls. Results are presented as mean ± SD of triplicate samples. *p < 0.05.

Following the observation that splenic, lung parenchymal and BAL CD11c+ cells were able to activate allogeneic T cells differentially, we next examined whether these APC populations differed in their capacity to activate naïve antigen-specific transgenic T cells. To this end, CD8 T cells from the TCR transgenic OT-I mice were purified and labeled with CFSE and then co-cultured with CD11c+ cell populations purified from the BAL, lung parenchyma, and spleen which were prior pulsed with CD8+ T cell OVA peptide SIINFEKL. We found that BAL APCs (1.6%) were the poorest naïve CD8+ T cell stimulators compared to lung parenchymal (7.8%) and splenic APCs (29.8%) (Figure 4A). To examine transgenic CD4 T cell responses, APCs from the BAL, lung interstitium and spleen were pulsed with a CD4 T cell OVA peptide before co-culturing with OT-II transgenic CD4 T cells in vitro. Again, the BAL APCs stimulated only a lower level of OT-II CD4 T cell proliferation (13.9%) than lung interstitial APCs whereas splenic APCs stimulated the highest level of OT-II T cell responses (47.4%) (Figure 4B).

### Differential levels of naïve antigen-specific transgenic T cell activation by virally-infected CD11c+ APC populations from the alveolar space, lung parenchyma and spleen

**Figure 5:**
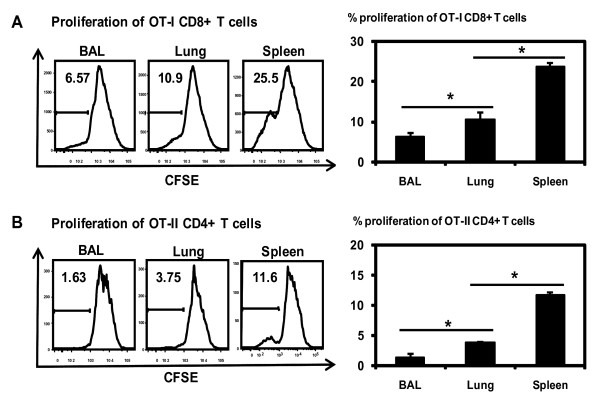
**Antigen-specific transgenic T cell activation stimulated by virally transduced CD11c+ APCs from BAL, lung parenchyma and spleen.** CD11c+ APC populations isolated from BAL, lung and spleen were infected with AdOVA and co-cultured either for 48 hours with CFSE-labelled OT-I CD8 T cells or for 72 hours with CFSE-labelled OT-II CD4 T cells. The rate of transgenic T cell proliferation was analyzed by FACS. A, OT-I CD8 T cell proliferation. B, OT-II CD4 T cell proliferation. Representative histograms of T cell proliferation are shown. The average T cell proliferation rates are shown in bar graphs after subtracting from appropriate controls. Results are presented as the mean ± SD of triplicate samples. *p < 0.05.

Since the above approach utilized antigenic peptides to directly load APCs, it represents a rather innocuous, artificial way to prepare APCs for antigen presentation and T cell activation. To further evaluate and compare antigen processing and presentation and T cell activation by CD11c+ APC populations from the BAL, lung parenchyma and the spleen, we set out to infect CD11c+ APC populations by using a recombinant adenovirus expressing OVA, a process that would involve natural infection, intracellular antigen processing and MHC pathway targeting and subsequent antigen presentation. To this end, CD8+ T cells from OT-I transgenic mice and CD4+ T cells from OT-II transgenic mice were purified and CFSE-labeled and then co-cultured with varying numbers of APCs from the BAL, lung, and the spleen while keeping the T cell number the same. A ratio of 1:2 of APCs vs. T cells elicited the optimal amount of proliferation in our system. Compared to the direct peptide loading approach, virally transduced APCs in general stimulated a lower level of transgenic CD8 and CD4 T responses (Figure 5A/B). However, the T cell stimulation profile by virally transduced APCs from the BAL, lung parenchyma and spleen recapitulated that by peptide-loaded APCs (Figures 4A/B and 5A/B). These results thus together reaffirm our above observations that BAL CD11c+ cells have the weakest ability to activate naïve antigen specific T cells compared to their lung parenchymal and splenic counterparts.

### Alveolar space CD11c+ APCs, similar to lung interstitial and splenic APCs, can lead to potent antigen recall responses of in vivo primed endogenous T cells

**Figure 6:**
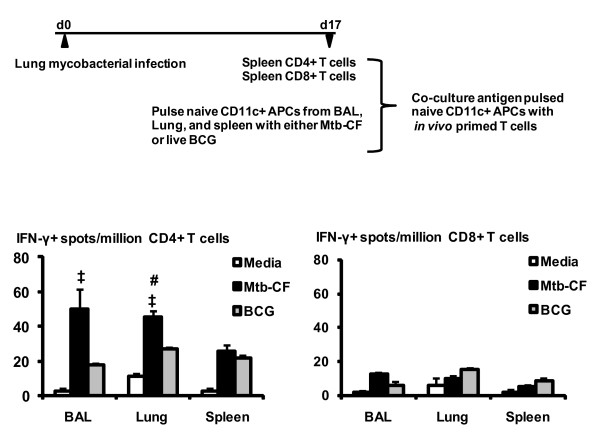
**Ex vivo activation of in vivo mycobacterium-primed natural T cells by CD11c+ APCs from BAL, lung parenchyma and spleen.** CD11c+ APC populations were isolated from BAL, lung parenchyma and the spleen and pulsed with M.tb CF protein or infected with live mycobacteria. APCs were then co-cultured with CD4+ or CD8+ T cells that were purified from the mice that were infected by live mycobacteria for 17 days (diagram). Cells were co-cultured for 24 h and IFN-γ-secreting T cells were determined by ELISPOT assay. A, IFN-γ-secreting CD4+ T cells. B, IFN-γ-secreting CD8+ T cells. Data are expressed as the mean value ± SD of triplicate samples and representative of two independent experiments. ‡p < 0.05 compared to the corresponding mycobacterial BCG-infected APCs; #p < 0.05 compared to the corresponding spleen data.

The above approaches only allowed us to analyze the responses of naïve transgenic CD4+ and CD8+ T cells stimulated by CD11c+ APCs in vitro. To investigate whether APC populations from the three different compartments may differ in their capability to activate the natural T cells that were spontaneously activated during the course of infection, we compared the CD11c+ cell populations from the BAL, lung parenchyma and the spleen for their ability to re-activate mycobacterial antigen-specific, IFN-γ-releasing type 1 T cells that naturally developed in vivo during mycobacterial infection. This is a particularly relevant question given that the alveolar space APCs largely made up by macrophages are the primary cellular targets of mycobacterial infection in the lung [42] and that these cells were previously shown to be able to activate certain T cells in mycobacterial infection [9]. To this end, mice were infected via the airway with live mycobacterial BCG and in vivo primed CD4+ and CD8+ T cells were isolated from the spleen. These T cells were then co-cultured with the CD11c+ cells freshly isolated from naïve mouse alveolar space, lung parenchyma, and spleen that were either soluble mycobacterial antigen-loaded or live mycobacterium-infected in vitro (Figure 6 diagram). Mycobacterial antigen-specific recall activation of T cells was measured by evaluating IFN-γ secreting cells in an ELISPOT assay. Contrast to their inability to markedly activate naïve transgenic T cells, BAL CD11c+ APCs activated T cells, particularly CD4 T cells, to a similar extent as the lung parenchymal and splenic APCs (Figure 6A/B). In general, soluble mycobacterial antigen loading of APCs led to higher levels of CD4 T cell activation than live mycobacterial infection. We observed a greater frequency of antigen-specific type 1 CD4 T cells than CD8+ T cells (Figure 6A/B), which is in agreement with the current understanding that mycobacterial infection activates predominantly a type 1 CD4 T cell response [43]. These data suggest that alveolar CD11c+ APCs can activate antigen experienced T cells to a similar extent as lung parenchymal and splenic CD11c+ APCs.

## Discussion

Antigen presenting cells play a pivotal role in initiating T cell responses against foreign pathogens. Their role becomes even more important at mucosal sites of the body including the skin, gut and lung where maximal microbial encounter occurs. Despite the recognized importance of APCs in eliciting T cell responses in the lung, our understanding regarding the relative T cell activation capacity of alveolar space (BAL), lung parenchymal, and splenic CD11c+ APCs still remains largely to be understood due to the lack of side-by-side comparative studies. In the present study we have evaluated and compared the phenotype of BAL, lung parenchymal, and splenic CD11c+ APC populations and their capabilities to activate both naïve and antigen-primed T cells. We report here that CD11c+ APCs from the BAL, lung parenchyma, and the spleen display differential co-stimulatory molecule expression and cytokine responsiveness. Furthermore, BAL CD11c+ APCs are incapable of markedly activating naïve T cells in contrast to lung parenchymal and splenic CD11c+ APC populations. However, these alveolar space CD11c+ APCs are able to potently activate the in vivo microbial antigen-primed T cells to a similar extent as lung parenchymal and splenic CD11c+ APCs.

The surface marker CD11c has been widely used for identifying and/or purifying antigen presenting DCs and macrophages [22,35-40,44] although it may also be expressed by other leukocyte subsets [37]. We believe that the majority of alveolar space CD11c+ APCs isolated by lavaging naïve mouse lung we used in our study are alveolar macrophages, different from the BAL of infected lungs which may contain heterogenous CD11c+ APC populations. This conviction is also supported by other published studies demonstrating that the vast majority (> 96%) of BAL CD11c+ cells are alveolar macrophages [39,40]. However, it is likely that a small fraction of these naïve BAL CD11c+ APCs may be DCs as it has previously been shown that a very minute fraction (< 2%) of BAL CD11c+ APCs are DCs [39]. On the other hand, the lung interstitial CD11c+ APCs used in this study are most likely a mixture of pulmonary DCs and macrophages. It is noteworthy that although the use of additional surface markers such as F4/80 and DEC-205 in conjunction with CD11c may aid in differentiating macrophages from DCs [22,35-40,44], it has been reported that F4/80 can also be expressed by DCs [37] and that DEC205 can also be expressed by pulmonary macrophages [45]. Our findings highlight that the tissue microenvironment or the anatomical location influences the function of APCs. Von Garnier et al. have shown that dendritic cells within the main conducting airway tissue (the trachea and main bronchi) and the lung parenchyma differ in their capacity to activate OVA-specific CD4+ T cell activation [5]. Similarly, we have shown that the CD11c+ APCs from the alveolar space are weak allogenic stimulators and are incapable of activating naive OVA-specific CD4+ and CD8+ T cells contrast to lung parenchymal and splenic CD11c+ APCs. However, we demonstrate that alveolar space CD11c+ APCs were able to activate antigen-primed type 1 CD4+ and CD8+ T cells to a similar extent as the lung parenchymal and splenic CD11c+ APCs. To our knowledge, our current study is the first to have directly compared alveolar space, lung parenchymal, and splenic CD11c+ APCs for their phenotypes and capacities to activate both naïve and primed T cells and concluded that alveolar space or BAL CD11c+ APCs are much poorer activators of naïve T cells than those isolated from the lung parenchyma and the spleen. This observation is in general agreement with the finding that alveolar macrophages may be immunosuppressive [25]. The fact that contrast to the relatively strong naïve T cell-activating capability of lung parenchymal and splenic CD11c+ APCs, BAL CD11c+ APCs is a poor activator of naïve T cells, suggests that a small fraction of DCs, if any, present in the BAL CD11c+ APC population plays a negligible role. However, we cannot completely rule out the possibility that under steady state conditions (naïve), the T cell activating capacity of contaminating DCs (if any) may be suppressed by the alveolar macrophages and with antigen-primed T cells, these BAL DCs may overcome the inhibitory effect of the alveolar macrophages to more readily activate antigen-primed T cells which may have decreased activation threshold. However, we feel that this is unlikely since the lung interstitial or splenic CD11c+ APCs activated antigen-primed T cells to a similar extent as they did to naïve T cells. It has also been demonstrated that alveolar macrophages can indeed act as accessory cells for mycobacterial antigen experienced γδ T cells [9]. Our results suggest that naïve T cells and antigen-primed effector T cells may have differential activation requirements. Furthermore, our results imply that alveolar macrophages and lung interstitial APCs play a distinct role in the early phase of T cell priming and activation with the latter playing the most critical role. However, during the effector phase of T cell responses, both alveolar macrophages and lung APCs could go on to present antigens and activate antigen-primed effector T cells. Given the large number of alveolar macrophages at the site of infection, these APCs may play an even greater role than DCs in effector T cell activation within the respiratory tract. We have recently demonstrated that airway luminal T cells are critical to immune protection against intracellular bacterial infection such as pulmonary tuberculosis [46,47].

Moreover, the ability of APCs to activate T cells is related to their co-stimulatory molecules expression as demonstrated by studies in which T cell proliferation is strongly inhibited by abrogation of either CD80 (B7.1) or CD86 [48,49], signifying that CD11c+ cell populations from the alveolar space, lung parenchyma, and the spleen all have the capacity to activate T cells since they all express MHC II and the co-stimulatory molecules B7.1 and CD40, albeit at different levels. This may explain the differential activation of naïve T cells by BAL, lung parenchymal and splenic CD11c+ APCs. In general, we observed relatively robust responses of both transgenic CD8 and CD4 T cell responses to the APCs loaded with OVA peptides. This is likely due to the efficiency of direct MHC loading and presentation of OVA peptides, particularly the CD8 T cell peptide. In comparison, the transgenic CD4 T cell responses to the APCs infected with an adenovirus expressing OVA protein were considerably lower than CD8 T cell responses. This is in accord with the understanding that virus infection preferentially target the MHC I pathway (CD8 T cell activation). This observation was noted across all three CD11c+ APC populations, suggesting that despite coming from different anatomical sites, these CD11c+ APC populations process viral antigens in a similar manner for naïve T cell activation. We have also reported here that freshly isolated BAL CD11c+ cells produce significantly greater levels of the pro-inflammatory cytokine TNF-α than their parenchymal and splenic counterparts, which is in agreement with the current understanding that alveolar macrophages are a great source of TNF-α [50]. However, parenchymal CD11c+ APCs produce a greater amount of the type 1 cytokine IL-12 compared to BAL CD11c+ APCs, which is in agreement with the stronger ability of the parenchymal CD11c+ APCs to activate naïve T cells. This disparate levels of TNF-α and IL-12 released by BAL and lung parenchymal CD11c+ APCs to LPS or mycobacterial antigens may be due to their use of distinct TLRs and differential TLR expression [29,51,52], which is likely influenced by the different tissue microenvironments [53,54]. IL-10, a potent Th1 opposing cytokine, was undetectable in our CD11c+ APC cultures from all the three compartments, suggesting that this cytokine does not account for the differential type 1 cytokine responses seen in our study. Our study reinforces that the knowledge generated by using APCs from one tissue site cannot be generalized and applied for all due to the heterogeneity of APCs tailored to different anatomic locations and specialized functions.

In conclusion, we have provided the evidence that the CD11c+ APC populations existing within the alveolar space, lung parenchyma, and the spleen differ much in their co-stimulatory molecule expression, cytokine responsiveness, and antigen presentation and T cell activation capacities. Furthermore, we have shown that alveolar space macrophages are poor activators of naïve T cells but can activate antigen experienced T cells to a similar extent as lung parenchymal and splenic CD11c+ APC populations. The findings presented in this study thus enhance our understanding about the relative capacity of alveolar space, lung parenchymal and splenic CD11c+ APCs to activate T cells, and further highlights the importance of tissue microenvironment influencing antigen presenting function of different APC populations.

## Conclusion

Our study suggests that alveolar CD11c+ APCs have a specialized T cell-activating function, capable of activating antigen-primed, but not naïve, T cells whereas lung CD11c+ APCs are capable of activating both the naïve and antigen-primed T cell populations.

## Methods

### Mice

Six- to 10-wk-old female C57Bl/6 and Balb/c mice were purchased from Harlan Laboratories. Female/male OT-I and OT-II transgenic mice were bred at McMaster University Central Animal Facility. All mice were housed in a specific pathogen-free level B facility. All experiments were conducted in accordance with the McMaster Animal Research Ethics board.

### Mycobacterial preparation

*Mycobacterium bovis *BCG (Connaught strain) was prepared as previously described in our lab [55,56]. Briefly, BCG was grown in Middlebrook 7H9 broth (Difco) supplemented with Middlebrook OADC enrichment (Invitrogen), 20% glycerol, and 0.05% Tween 80 for 10 to 15 days, and samples were then divided into aliquots and stored at -70°C. BCG was washed twice with phosphate-buffered saline (PBS) containing 0.05% Tween 80 and resuspended in PBS. It was then passed through a 27-gauge needle 10 times to disperse clumps and then diluted with PBS to the desired concentration before use.

### Isolation of CD11c+ APCs from the alveolar space, lung parenchyma, and spleen

Naïve mice were sacrificed by bleeding the abdominal vessels. Mouse lungs were then removed aseptically and lavaged with PBS to isolate bronchoalveolar lavage (BAL) cells from the alveolar space. The mouse lung was exhaustively lavaged 5 times to a total volume of 1.8 ml PBS through a polyethylene cannulated into the trachea to ensure maximal cell recovery. Extra effort was made to ensure that only the lung parenchyma was collected and any trachea and main bronchi including all associated hilar lymph nodes were removed. The lavaged lungs and the spleens were infused with collagenase type 1 (Sigma) and then cut up into small pieces and subsequently incubated in collagenase at 37°C; 1 hour for lungs and 45 minutes for spleens. The collagenase digested lungs and spleens were then passed through 100 μm cell strainers, and a 3 ml needle plunger was used to mash the cells through the strainer. After red blood cell lysis with a mouse erythrocyte lysing kit (R&D Systems), lungs and spleens were filtered and resuspended in complete RPMI (cRPMI) media (RPMI 1640 supplemented with 10% fetal bovine serum, 1% penicillin-streptomycin, 1% L-glutamine). Cells were counted, and viability (always > 90%) was measured by trypan blue exclusion. Depending on the experiment, BAL, lung, and spleen cells from a number of mice were pooled and incubated with CD11c microbeads (Miltenyi biotec) according to the manufacturer's instructions. CD11c labelled cells were then passed through an MS column on the OctoMACS separator (Miltenyi biotec). Samples were run through MACS separation columns twice to achieve higher purity. Cells were counted, and viability was measured by trypan blue exclusion. Purity of cell preparations were determined using flow cytometry, and the purity of CD11c+ populations was consistently > 90%.

### Cell surface immunostaining and FACS analysis

All monoclonal antibodies (MAbs) used were purchased from BD Pharmingen. Immunostaining and FACS were carried out as previously described [55,56]. Briefly, cells were blocked for non-specific binding of their Fc receptors with anti-CD16/CD32 antibodies for 15 min and then stained for 30 min on ice with the appropriate combinations of fluorochrome-conjugated MAbs. Fluorochrome-conjugated MAbs to CD11c, B7.1, CD40, MHC Class II, CD3, CD4, and CD8 were used. Appropriate Isotype controls were used for each antibody. The data was collected with the LSRII (BD Biosciences) flow cytometer using FACSDiva software and analyzed with Flowjo software.

### Cell culture and ELISA

Purified CD11c+ APCs (0.1 × 10^6^/well) from the alveolar space, lung parenchyma, and spleen were seeded into 96-well flat bottom plate and cultured at 37°C and 5% CO_2 _with or without antigen stimulation. The antigens used for stimulation were LPS (1 ng/well) and *Mycobacterium tuberculosis *Culture Filtrate proteins (Mtb-CF) (2 μg/well). Cells were cultured in a total volume of 250 μl of cRPMI. The culture supernatants were collected at 48 h and stored at -20°C until cytokine measurement. TNF-alpha and IL-12p40 concentrations were measured by using ELISA kits (R&D systems).

### Mixed leukocyte reaction (MLR)

MLR was carried out as previously described [53]. Allogeneic splenic T cells were isolated from naïve Balb/c mice using CD4 and CD8 positive isolation kits (both from Miltenyi biotec) according to the manufacturer's protocol and pooled. T cells were then labelled with 5 μM CFSE (Molecular Probes) in PBS supplemented with 5% FBS and then washed three times after staining. 5 × 10^5 ^CFSE labelled T cells were co-cultured with a varying number of C57Bl/6 CD11c+ APC populations from the alveolar space, lung parenchyma, and spleen in 96-well culture plate at 37°C for 96 hours. T cell proliferation was examined by flow cytometric analysis of CFSE dilution as previously described [57].

### *In vitro *transgenic T cell proliferation Assay

CD4 T cells were MACS purified from OT-II transgenic mice and CD8 T cells were purified from OT-I transgenic mice using the CD4+ and CD8+ T cell isolation kits, respectively, according to the manufacturer's instructions (Miltenyi biotec). T cells were labelled with 5 μM CFSE (Molecular Probes) and washed 3 times with PBS supplemented with 5% FBS and resuspended in cRPMI. 5 × 10^5 ^CFSE labelled OT-II T cells were co-cultured with varying numbers of C57Bl/6 APC populations from BAL, lung, and spleen that were pre-pulsed with CD4 or CD8 OVA peptide (0.1 ng/μl) for 1 hour. Unpulsed CD11c+ cells co-cultured with T cells and T cells cultured with no CD11c+ APCs were set up in parallel as controls. CD8+ and CD4+ T cell proliferations were examined 48 and 72 hours after culture by flowcytometric analysis of CFSE dilution, respectively. In some experiments, APCs were pre-infected with an adenovirus expressing the OVA protein (AdOVA) (100 pfu/cell) overnight and then co-cultured with OT-I or OT-II T cells. An adenoviral vector (Addl70-3) infected CD11c+ cells and uninfected CD11c+ cells were used as controls in these experiments.

### Ex vivo T cell activation assay

In vivo mycobacterium-primed CD4+ T cells and CD8+ T cells were MACS-purified as described above from the spleens of mice that had been infected with live *M. bovis *BCG (10^6 ^cfu) for 17d as previously described [55]. 0.4 × 10^6 ^T cells were co-cultured with 4000 naïve BAL, lung, and spleen CD11c+ APC populations that were infected with either BCG (2 cfu/cell) or stimulated with Mtb-CF (2 ug/well) or just culture media (no stimulus). IFN-γ secretion ELISPOT was carried out as previously described [58]. Briefly, isolated CD4+ and CD8+ T cells (0.4 × 10^6^) were seeded into a 96-well PVDF microplate (Millipore Corporation) pre-coated overnight with a mouse IFN-γ capture antibody (R&D system). Cells were incubated for 24 h and then washed and incubated with a detection antibody at 4°C overnight. The plate was developed by using standardized streptavidin-conjugated alkaline phosphatase and chromogen method (R&D system). The number of IFN-γ-releasing cells was determined by using an ELISPOT reader (CTL Cellular Technology Ltd).

### Statistical analysis

All statistical analyses were performed using unpaired, two-tailed student's *t *test with Excel spreadsheet software (Microsoft). Values of p < 0.05 were considered statistically significant.

## Authors' contributions

KK carried out and designed the experiments and wrote the manuscript. EKR helped with experiments and data analysis. C–LS assisted in infecting the mice and sample collections. SM helped with FACS data analysis and interpretation. PY provided invaluable advice and protocols on APC purification, staining and phenotyping. ZX crafted the idea of this project and contributed to the overall design and execution of experiments and helped with manuscript drafting and fine-tuning. All authors have read and approved the manuscript.
